# Influence of neglecting the curved path of the Achilles tendon on Achilles tendon length change at various ranges of motion

**DOI:** 10.14814/phy2.12176

**Published:** 2014-10-10

**Authors:** Atsuki Fukutani, Satoru Hashizume, Kazuki Kusumoto, Toshiyuki Kurihara

**Affiliations:** 1 Research Organization of Science and Technology, Ritsumeikan University, KusatsuShiga, Japan; 2 Japan Society for the Promotion of Science, Research Fellowship for Young Scientists, Chiyoda‐kuTokyo, Japan; 3 Faculty of Health and Sports Science, Juntendo University, InzaiChiba, Japan; 4 Faculty of Science and Industrial Technology, Kurashiki University of Science and the Arts, KurashikiOkayama, Japan; 5 Faculty of Sport and Health Science, Ritsumeikan University, KusatsuShiga, Japan

**Keywords:** Dorsiflexion, plantarflexion, three‐dimensional measurement, ultrasonography

## Abstract

Achilles tendon length has been measured using a straight‐line model. However, this model is associated with a greater measurement error compared with a curved‐line model. Therefore, we examined the influence of neglecting the curved path of the Achilles tendon on its length change at various ranges of motion. Ten male subjects participated in this study. First, the location of the Achilles tendon was confirmed by using ultrasonography, and markers were attached on the skin over the Achilles tendon path. Then, the three‐dimensional coordinates of each marker at dorsiflexion (DF) 15°, plantarflexion (PF) 0°, PF15°, and PF30° were obtained. Achilles tendon length in the curved‐line model was calculated as the sum of the distances among each marker. On the other hand, Achilles tendon length in the straight‐line model was calculated as the straight distance between the two most proximal and distal markers projected onto the sagittal plane. The difference of the Achilles tendon length change between curved‐line and straight‐line models was calculated by subtracting the Achilles tendon length change obtained in curved‐line model from that obtained in straight‐line model with three different ranges of motion (i.e., PF0°, PF15°, and PF30° from DF15°, respectively). As a result, the difference in Achilles tendon length change between the two models increased significantly as the range of motion increased. In conclusion, neglecting the curved path of the Achilles tendon induces substantial overestimation of its length change when the extent of ankle joint angle change is large.

## Abstract (precis)

e12176

This study examined the influence of neglecting the curved path of the Achilles tendon on its length change. As a result, neglecting the curved path of the Achilles tendon induces substantial overestimation of its length change when the extent of ankle joint angle change is large.

## Introduction

The Achilles tendon is believed to play an important role in dynamic movements, such as walking and jumping, due to its ability to store and release elastic energy during these movements (Fukashiro et al. 1995; Magnusson et al. 2008). Therefore, many studies have evaluated Achilles tendon length change during dynamic movements. For example, Fukunaga et al. (2001) examined the behavior of the Achilles tendon during walking, and suggested that its spring‐like behavior would improve the energy cost of walking. In addition, Kurokawa et al. (2001) reported that Achilles tendon compliance would contribute to optimizing muscle behavior for generating force during jumping.

To examine the effect of Achilles tendon length change on the aforementioned human movements, accurate measurement is required. However, because direct measurement of Achilles tendon length change is difficult, especially during dynamic movements, indirect measurement has been applied instead in previous studies. For example, Fukunaga et al. (2001) and Ishikawa et al. (2007) adopted an estimation equation for calculating Achilles tendon length change. In concrete terms, Achilles tendon length change has been estimated by subtracting muscle (fascicle) length change obtained by using ultrasonography from muscle‐tendon complex length change estimated by using joint angle change (Grieve et al. 1978; Hawkins and Hull 1990). On the other hand, Hoffrén et al. (2012) tried to evaluate Achilles tendon length change during repetitive hopping without using an estimation equation, by determining the coordinates of both ends of the Achilles tendon; coordinate of proximal end was determined by ultrasonographic image and coordinate of distal end was determined by video images of the reflective marker attached on the skin.

Essentially, however, the above methods do not consider the curved shape of the Achilles tendon. For example, since Hoffrén et al. (2012) adopted a straight‐line model, the curvature of the Achilles tendon was not considered. Thus, its length was inevitably underestimated. In this regard, Stosic and Finni (2011) examined the influence of tendon length measurement obtained by using a straight‐line model on strain of the Achilles tendon during hopping by comparing Achilles tendon length change obtained by using a curved‐line model. As a result, strain of the Achilles tendon was 1.3% larger in the straight‐line model compared with that in the curved‐line model. The influence of this overestimation on Achilles tendon length change may be small. However, the result of Stosic and Finni (2011) did not consider the change in the location of the proximal end of the Achilles tendon; that is, they assumed that the proximal end of the Achilles tendon does not move by muscle contractions and/or joint angle changes. In addition, range of motion in their study was limited, probably only in the plantarflexion (PF) region, because they adopted ankle hopping with knee joint angle constant on the force platform. Considering that the magnitude of curvature of the Achilles tendon may vary between dorsiflexion (DF) and PF regions, the influence of neglecting the curved path may be different when the range of motion is different. Taking these into account, further study is required.

Therefore, the purpose of this study was to examine the influence of neglecting the curved path of the Achilles tendon on its length change at various ranges of motion by calculating the difference in Achilles tendon length change between a straight‐line model and a curved‐line model. In addition, to obtain better understanding of Achilles tendon length change obtained by using an estimation equation that has been widely applied in previous studies (Fukunaga et al. 2001), Achilles tendon length change obtained by using a curved‐line model was compared with that obtained by using the estimation equation.

## Materials and Methods

### Subjects

Ten healthy male volunteers (age, 20.2 ± 1.2 years; height, 1.70 ± 0.05 m; body mass, 61.7 ± 3.7 kg) were recruited for this study. The purpose and risks of this study were explained to each volunteer, and written informed consent was obtained. The Ethics Committee on Human Research of Ritsumeikan University approved this study (IRB‐2012‐030).

### Calculation of Achilles tendon length change

Achilles tendon length was measured at DF15°, PF0°, PF15°, and PF30° during relaxed conditions. Ankle joint angle was controlled by using a myodynamometer (Biodex; SAKAImed, Tokyo, Japan). Center of rotation of the ankle joint was carefully confirmed in accordance with the center of rotation of the myodynamometer. Throughout the experiments, knee and hip joint angles were fixed at 0 and 80° (knee and hip joint angles at their anatomic positions were defined as 0°), respectively.

**Figure 1.**
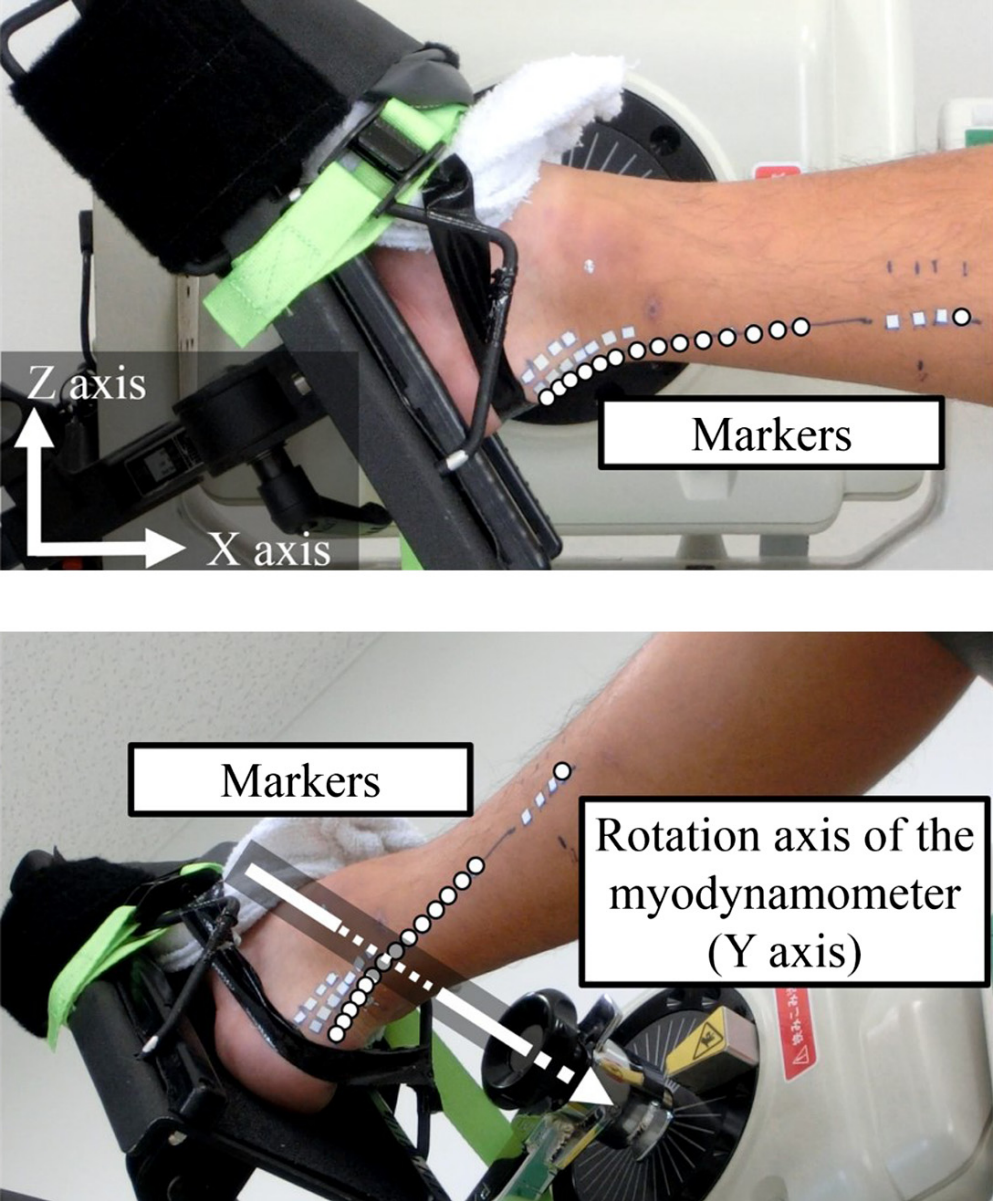
Experimental setting for calculating Achilles tendon length change. Two synchronized video images were used to obtain the three‐dimensional coordinates of each marker (white circles). The upper figure shows the lateral view; the lower figure shows the inferior view. The *X*‐, *Y*‐, and *Z*‐axes are indicated by white arrows. The axis of rotation of the myodynamometer was defined as the *Y*‐axis, the vertical line was defined as the *Z*‐axis, and the *X*‐axis was perpendicular to the *Y*–*Z* plane.

To obtain Achilles tendon length, three‐dimensional (3D) coordinates of markers attached on the skin along the Achilles tendon were calculated. In this surface marker method, free Achilles tendon length (i.e., from the distal end of the medial gastrocnemius to the calcaneal tuberosity) was evaluated. First, both ends of the Achilles tendon were identified by using ultrasonography (SSD‐3500; Aloka, Tokyo, Japan) at DF15°, PF0°, PF15°, and PF30°. Proximal and distal ends of the Achilles tendon were defined as the muscle‐tendon junction of the medial gastrocnemius and the calcaneal tuberosity, respectively. Then, markers were attached to the skin along the Achilles tendon path, and the 3D coordinates of the markers were obtained using two video cameras (EX‐ZR400; CASIO, Tokyo, Japan) (Fig. 1). Definitions of the right‐handed orthogonal coordinate system were as follows: the *Y*‐axis was parallel to the rotation axis of the myodynamometer, the *Z*‐axis was the vertical axis, and the *X*‐axis was perpendicular to the *Y*–*Z* plane (Fig. 1). In the current study, the *Z*–*X* plane was assumed to be the sagittal plane. In the 3D curved‐line model (3D‐curve), Achilles tendon length was calculated as the sum of the 3D distances between adjacent markers. On the other hand, Achilles tendon length in the two‐dimensional (2D) straight‐line model (2D‐straight) was calculated as the straight distance between the markers attached to both ends of the Achilles tendon projected onto the sagittal plane. The difference in Achilles tendon length was calculated by subtracting the value obtained by using 3D‐curve from that obtained by using 2D‐straight.

In addition, Achilles tendon length change was calculated by using a 3D straight‐line model (3D‐straight) and a 2D curved‐line model (2D‐curve) in which the ankle joint angle moved from DF15° to PF30°. In 3D‐straight, Achilles tendon length was calculated from the 3D coordinates of two makers attached to both ends of the Achilles tendon. The straight distance between these two markers was defined as Achilles tendon length. In 2D‐curve, Achilles tendon length was calculated as the sum of the distances among the 2D coordinates of all markers projected onto the sagittal plane.

In a preliminary study, we confirmed the validity of calculating Achilles tendon length from the coordinates of surface markers, by comparing data obtained using a magnetic resonance imaging (MRI) method with the following parameters: fast spin echo; echo time, 21.2 msec; repetition time, 1400 msec; slice thickness, 1 mm; interslice distance, 1 mm; field of view, 360 × 360 mm; and matrix, 512 × 512 pixels (voxel resolution, 0.70 × 0.70 × 1 mm). Achilles tendon length in the MRI method was calculated as the sum of the distances among the 3D coordinates of the digitized points of the Achilles tendon from the calcaneal tuberosity to the medial gastrocnemius‐Achilles tendon junction. Achilles tendon length was measured at three ankle joint angles (DF20°, PF0°, and PF20°) by using an identical method in the current study (3D‐curve) and an MRI method in eight subjects. As a result, there was no significant difference between the two methods (marker, 181.7 ± 16.4 mm; MRI, 183.3 ± 17.9 mm; *P *<**0.077), and the coefficient of correlation was 0.973.

### Additional calculation of Achilles tendon length change with estimation equation

Achilles tendon length change was also calculated by using an estimation equation (Fukunaga et al. 2001; Kurokawa et al. 2001; Ishikawa et al. 2007), which has been widely used in previous studies, to compare Achilles tendon length change calculated by using 3D‐curve. In this estimation equation method, not only free Achilles tendon length change but also aponeurosis length change was included unlike surface marker method. Achilles tendon length change was determined by using the identical method of Fukunaga et al. (2001). Briefly, length change in the medial gastrocnemius fascicle was subtracted from length change in the triceps surae muscle‐tendon complex. The muscle‐tendon complex length change was estimated by using ankle and knee joint angles and lower leg length (Grieve et al. 1978). In the present study, the knee joint angle was fixed at 0° throughout the experiments. Medial gastrocnemius fascicle length was measured directly by using longitudinal ultrasonographic images at DF15° and PF30°, and was corrected by considering pennation angle (i.e., fascicle length × cos*α*). Lower leg length, defined as the straight distance between the knee crease and the lateral malleolus, was measured using a steel measure (KDS F10‐02; Muratec, Tokyo, Japan) in increments of 5 mm.

### Statistical analysis

One‐way analysis of variance (ANOVA) with repeated measures was conducted to compare the magnitude of difference in Achilles tendon length change between 3D‐curve and 2D‐straight at various ranges of motion. If a significant main effect was found, an additional post hoc test (paired *t* test with Bonferroni correction) was performed.

For Achilles tendon length change calculated by using 3D‐curve, 3D‐straight, 2D‐curve, and 2D‐straight when the ankle joint moved from DF15° to PF30°, one‐way ANOVA with repeated measures was conducted to compare the magnitude of Achilles tendon length change among the four different methods. If a significant main effect was found, an additional post hoc test (paired *t* test with Bonferroni correction) was performed.

Pearson's product–moment correlation coefficient analysis was conducted to examine the relationship between Achilles tendon length change calculated by using 3D‐curve and that calculated by using the estimation equation.

Descriptive data are presented as mean ± SD. The level of statistical significance was set at *P *<**0.05. Statistical analyses were performed using SPSS version 20 software (IBM, Tokyo, Japan).

## Results

In 3D‐curve, the Achilles tendon lengths at DF15°, PF0°, PF15°, and PF30° were 188.9 ± 30.2 mm, 187.9 ± 31.0 mm, 186.4 ± 29.7 mm, and 183.3 ± 26.5 mm, respectively. In 2D‐straight, the Achilles tendon lengths at DF15°, PF0°, PF15°, and PF30° were 187.6 ± 29.8 mm, 186.4 ± 30.6 mm, 183.5 ± 29.2 mm, and 178.7 ± 25.4 mm, respectively.

**Figure 2.**
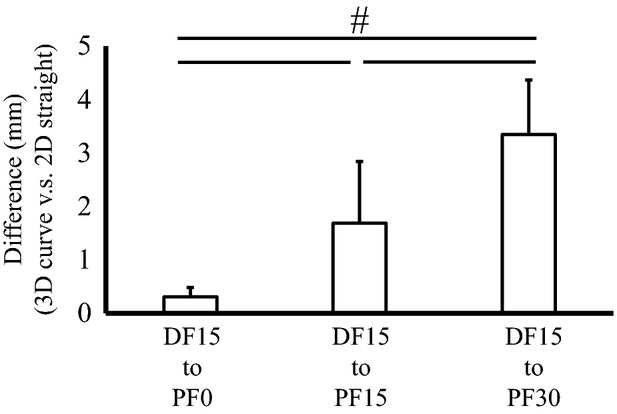
Difference in Achilles tendon length change calculated by using a three‐dimensional curved‐line model (3D‐curve) and a two‐dimensional straight‐line model (2D‐straight). ^#^indicates a significant difference between ranges of motion (*P *<**0.05).

Figure 2 shows the magnitude of difference in Achilles tendon length change obtained between 3D‐curve and 2D‐straight. A significant main effect was found (*F *=**91.147, effect size = 0.91, *P *<**0.001) and the post hoc test revealed that the magnitude of difference was significant among ranges of motion, which was largest in the DF15° to PF30° condition and smallest in the DF15° to PF0° condition.

**Figure 3.**
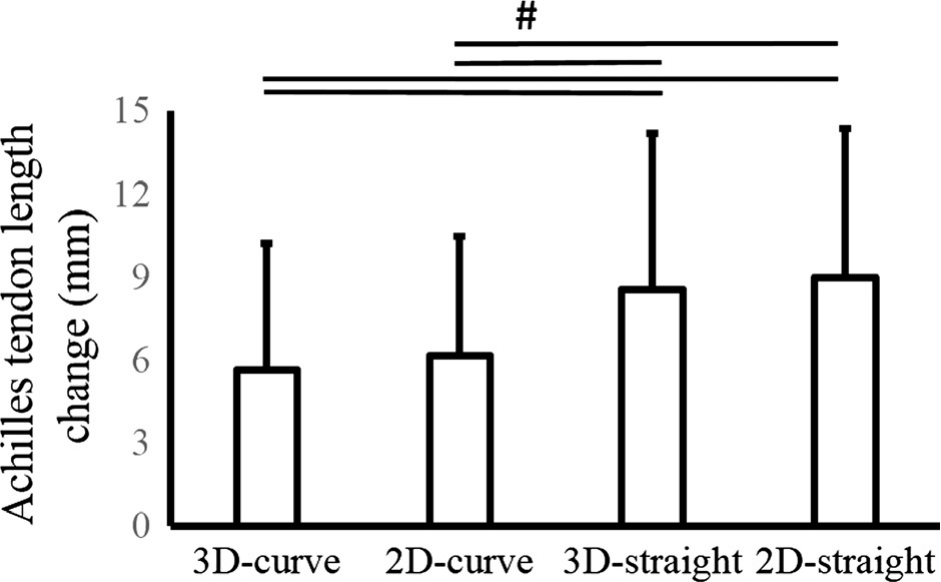
Achilles tendon length change using four different methods. Achilles tendon length change was calculated when the ankle joint moved from DF15° to PF30° using a three‐dimensional (3D) curved‐line model (3D‐curve), 3D straight‐line model (3D‐straight), two‐dimensional (2D) straight‐line model (2D‐straight), and 2D curved‐line model (2D‐curve). ^#^indicates a significant difference between methods (*P *<**0.05).

Figure 3 shows the magnitude of Achilles tendon length change obtained by using the four different surface marker methods. A significant main effect was found (*F *=**46.580, effect size = 0.84, *P *<**0.001). Achilles tendon length change was larger in 2D‐straight and 3D‐straight than in 3D‐curve and 2D‐curve (*P *<**0.001). On the other hand, Achilles tendon length change was not different between 3D‐curve and 2D‐curve (*P *>**0.05).

**Figure 4.**
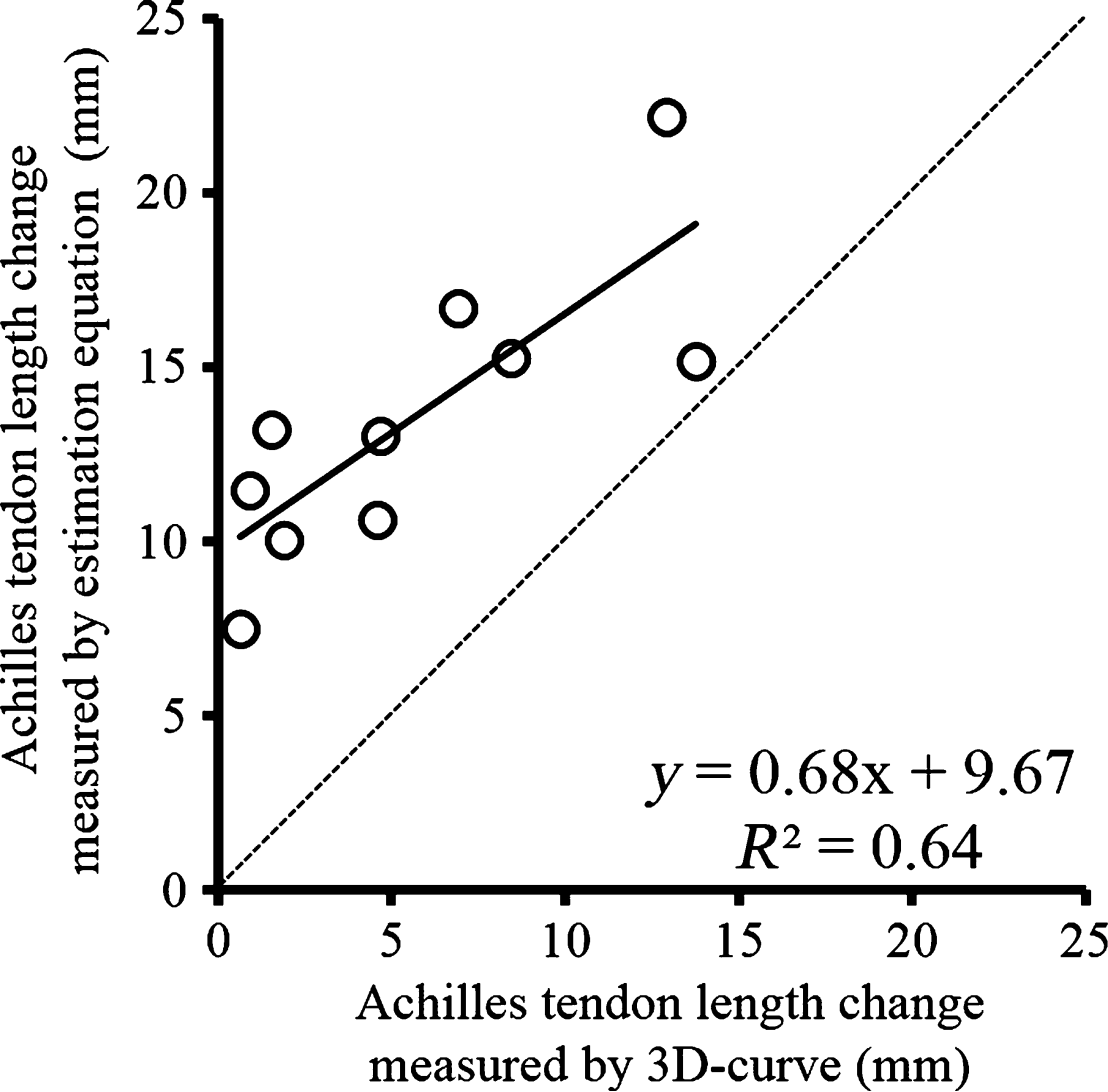
Correlation of Achilles tendon length change between a three‐dimensional curved‐line model (3D‐curve) and an estimation equation when the ankle joint moved from DF15° to PF30°. The dotted line shows the identical line (*X*‐axis = *Y*‐axis).

Figure 4 shows the significant correlation between Achilles tendon length change obtained between 3D‐curve and the estimation equation (*P *=**0.005); however, the coefficient of determination was not high (*R*^2^ = 0.64). All values obtained by using the estimation equation were larger than those obtained by using 3D‐curve.

## Discussion

This study examined the influence of neglecting the curved path of the Achilles tendon on its length change at various ranges of motion. The difference in Achilles tendon length change between 3D‐curve and 2D‐straight increased significantly as the range of motion increased. Considering the magnitude of Achilles tendon length change calculated using the 3D‐curve (5.6 ± 4.6 mm) and 2D‐straight (9.0 ± 5.4 mm) obtained in the DF15° to PF30° condition (Fig. 3, bar 1 and bar 4), the extent of error (78% larger in 2D‐straight) has a substantial influence on the interpretation of Achilles tendon length change.

The reason for the relatively large influence of neglecting the curved path of the Achilles tendon in the current study compared with that in a previous study (Arampatzis et al. 2008; Stosic and Finni 2011), may be attributed to the range of motion adopted. Previous studies adopted relatively small ranges of motion, and ranges of motion were limited within the PF region. Especially, Arampatzis et al. 2008 adopted isometric contractions. On the other hand, the current study adopted a large range of motion including DF and PF regions. In the DF region (i.e., DF15°), the Achilles tendon is almost straight; thus Achilles tendon length was similar between 3D‐curve (188.9 ± 30.2 mm) and 2D‐straight (187.6 ± 29.8 mm). On the other hand, in the PF region (i.e., PF30°), in which the Achilles tendon is curved, Achilles tendon length was different between 3D‐curve (183.3 ± 26.5 mm) and 2D‐straight (178.7 ± 25.4 mm). Therefore, Achilles tendon length change is substantially overestimated when the ankle joint angle moves from DF to PF. In contrast, when the range of motion is limited, such as within the DF region (Fig. 2, bar 1), the influence of neglecting the curved path of the Achilles tendon is not large.

The influence of neglecting the curved path of the Achilles tendon on its length change was clearly illustrated by the results of the four different surface marker methods, calculated in the condition in which the largest difference in Achilles tendon length change was observed (i.e., DF15° to PF30°) (Fig. 2, bar 3). In 2D‐straight and 3D‐straight, which ignored the tendon's curved path (Fig. 3, bar 3 and 4), Achilles tendon length change was larger than that obtained in 3D‐curve and 2D‐curve, which considered the tendon's curved path (Fig. 3, bar 1 and 2). These results show that if the curved path of the Achilles tendon is not considered, the magnitude of Achilles tendon length change is overestimated. On the other hand, if the curved path is taken into account, the value of Achilles tendon length change calculated by using 2D‐curve (projection onto the sagittal plane) would be acceptable as a simplified measurement, even in the condition in which a substantial influence of neglecting the curved path of the Achilles tendon is expected (Fig. 3, bar 1 and 2).

We compared Achilles tendon length change obtained by using 3D‐curve and by using an estimation equation that has been widely used in previous studies (Fukunaga et al. 2001; Hirayama et al. 2012). The results showed that Achilles tendon length change was clearly larger when using the estimation equation (13.5 ± 3.9 mm) compared with when using 3D‐curve (5.6 ± 4.6 mm), and the coefficient of determination was not large (*R*^2^ = 0.64) (Fig. 4). These results suggest that care should be given when interpreting Achilles tendon length change calculated by using the estimation equation. The observed differences between the two methods may be due to inaccurate estimation of length change of the triceps surae muscle‐tendon complex. This estimation equation method was established by Grieve et al. (1978) to estimate length change of the muscle‐tendon complex as a function of knee and ankle joint angle changes. To determine this equation, Grieve et al. (1978) cut the lower part of the Achilles tendon from cadaveric specimens, and measured the change in the “straight distance” of the cut portion when the ankle and knee joint angles were changed. Thus, this method would lead to overestimation of muscle‐tendon complex length change. As a result, Achilles tendon length change obtained by using this estimation equation was also overestimated.

Admittedly, we have to consider that Achilles tendon length change calculated by using this estimation equation includes not only free Achilles tendon (the distal side from the muscle‐tendon junction) length change but also aponeurosis length change, whereas that calculated by using 3D‐curve includes only free Achilles tendon length change. Thus, a larger value calculated by using the estimation equation may be reasonable. However, considering the fact that the subject who showed a large Achilles tendon length change calculated by using 3D‐curve did not necessarily show a large change calculated by using the estimation equation (Fig. 4), not only the absolute value but also the tendency of Achilles tendon length change should be different between the two calculation methods. This may be derived from an invalid assumption in the estimation equation. The estimation equation works well when fascicle length and pennation angle within the muscle behave uniformly. However, Shin et al. (2009) reported that changes in these parameters were nonuniform within muscle. Furthermore, the magnitude of strain of the free Achilles tendon and aponeurosis was reported to be different depending on the location within the muscle (Kaya et al. 2003), indicating that the tendency of Achilles tendon length change calculated by using the estimation equation (i.e., including free Achilles tendon and aponeurosis) does not necessarily represent the tendency of free Achilles tendon length change. Hence, care is needed when interpreting the physiologic meaning of Achilles tendon length change obtained by using the estimation equation.

## Conclusions

We conclude that the curved path of the Achilles tendon must be considered when Achilles tendon length change is evaluated. If the curved path is ignored, Achilles tendon length change is overestimated, especially when the ankle joint angle change is large. This phenomenon would result from the fact that the Achilles tendon length in the DF region calculated by using the straight‐line model is the same as that calculated by using the curved‐line model, even though the Achilles tendon length in the PF region calculated by using the straight‐line model is different from that calculated by using the curved‐line model.

## Acknowledgments

We thank the subjects for their time and dedication to our study.

## Conflict of Interest

There are no conflicts of interest for all authors.
